# Blood Transcriptomic Meta-analysis Identifies Dysregulation of Hemoglobin and Iron Metabolism in Parkinson’ Disease

**DOI:** 10.3389/fnagi.2017.00073

**Published:** 2017-03-29

**Authors:** Jose A. Santiago, Judith A. Potashkin

**Affiliations:** The Cellular and Molecular Pharmacology Department, The Chicago Medical School, Rosalind Franklin University of Medicine and Science, North ChicagoIL, USA

**Keywords:** iron metabolism, hemoglobin, Parkinson’s disease, neurodegeneration, meta analysis

## Abstract

Disrupted iron metabolism has been implicated in the pathogenesis of Parkinson’s disease (PD), a progressive neurodegenerative disorder that severely affects movement and coordination, yet the molecular mechanisms underlying this association remain unknown. To this end, we performed a transcriptomic meta-analysis of four blood microarrays in PD. We observed a significant downregulation of genes related to hemoglobin including, hemoglobin delta (*HBD*), alpha hemoglobin stabilizing protein (*ASHP*), genes implicated in iron metabolism including, solute carrier family 11 member 2 (*SLC11A2*), ferrochelatase (*FECH*), and erythrocyte-specific genes including erythrocyte membrane protein (*EPB42*), and 5′-aminolevulinate synthase 2 (*ALAS2*). Pathway and network analysis identified enrichment in processes related to mitochondrial membrane, oxygen transport, oxygen and heme binding, hemoglobin complex, erythrocyte development, tetrapyrrole metabolism and the spliceosome. Collectively, we identified a subnetwork of genes in blood that may provide a molecular explanation for the disrupted hemoglobin and iron metabolism in the pathogenesis of PD.

## Introduction

Parkinson’s disease (PD) is a devastating movement disorder that is estimated to affect 7–10 million people worldwide according to the PD Foundation^1^. Clinical characteristics of PD include bradykinesia, resting tremor, postural instability, and rigidity, which are caused by the progressive loss of dopaminergic neurons in the substantia nigra. To date, there is no effective therapy to halt the progression of the disease. A wide range of biological processes including, mitochondrial dysfunction, endoplasmic reticulum stress, inflammation, impaired insulin signaling, oxidative stress, and iron metabolism have been implicated in the pathogenesis of PD (Dawson and Dawson, 2003; Santiago and Potashkin, 2013b).

Disrupted iron homeostasis has been proposed to play a causative role in PD. This is not surprising, since iron plays a crucial role in vital cellular processes including, mitochondrial respiration, synthesis of myelin and neurotransmitters, nitric oxide metabolism and oxygen transport (Crichton et al., 2011; Schneider et al., 2012). In this context, perturbed iron metabolism has been shown to contribute to the generation of oxidative stress, alpha-synuclein (SNCA) accumulation, and dopaminergic cell death in PD (Salazar et al., 2008; Crichton et al., 2011; Matak et al., 2016). Increased iron deposits have been observed in brain regions of PD patients and the extent of its accumulation have been correlated with disease severity (Sofic et al., 1988; Gorell et al., 1995; Martin et al., 2008), reviewed in Rhodes and Ritz (2008). Further, excessive iron accumulation led to dopaminergic cell death through the production of reactive oxygen species in animal models of PD (Salazar et al., 2008; You et al., 2015). Strikingly, the A53T mutation in *SNCA*, central in the pathogenesis of PD, mediated iron accumulation and toxicity in neuroblastoma cells (Ostrerova-Golts et al., 2000).

Several studies suggest that a systemic iron deficiency, rather than an iron overload, may play an important role in the pathogenesis of PD. Earlier studies showed that serum levels of iron, ferritin, and transferrin were significantly lower in PD patients compared to healthy controls (HCs) (Logroscino et al., 1997). In support of this finding, the risk of PD was higher among men who reported multiple blood donations, an indicator of reduced iron stores (Logroscino et al., 2006). Interestingly, anemia, a condition characterized by low hemoglobin levels and sometimes associated with iron deficiency, has been correlated with an increased risk of PD in several populations (Savica et al., 2009; Hong et al., 2016). In addition, it has been proposed that hemoglobin may play a role in the oxidative stress and mitochondrial dysfunction in PD (Shephard et al., 2016).

Since high-throughput screening of RNA from blood has been instrumental in elucidating important molecular pathways underlying neurodegeneration in PD patients (Scherzer et al., 2007; Mutez et al., 2011; Potashkin et al., 2012; Alieva et al., 2014; Calligaris et al., 2015; Santiago et al., 2016; Simchovitz et al., 2016), we hypothesized that analysis of the blood transcriptome could provide clues for the disrupted iron metabolism observed in PD patients. To this end, we performed a meta-analysis of four independent blood microarrays from PD patients and HCs. We identified a network of downregulated genes related to hemoglobin and iron metabolism thus providing a molecular evidence for the impairment of these pathways in PD.

## Materials and Methods

### Microarray Meta-analysis Using NextBio

We used the curated database NextBio Research (Illumina, Inc., San Diego, CA, USA) (Kupershmidt et al., 2010) to search for gene expression studies in PD. Meta-analysis was performed according to the PRISMA guidelines (Moher et al., 2009) (**Supplementary Figure S1** and **Table S1**). Using the search terms “PD,” “blood,” “human,” “RNA” and “microarray,” we identified six studies in blood of PD. One study was a duplicate and therefore removed. Only human microarray studies curated in NextBio, including samples from sporadic PD and HCs were used in the analysis. One study involving patients with a *LRRK2* mutation was excluded from the analysis (GSE22491) since we focused on sporadic PD. In the final selection, four microarrays met our inclusion criteria. Description of microarray datasets included in this study is provided in **Table 1**. Microarray meta-analysis was perfomed in NextBio as described previously (Santiago et al., 2016). Briefly, differentially expressed genes were extracted from NextBio and negative values, if any, were replaced with the smallest positive number in the dataset. Genes whose mean normalized test and control intensities were both less than the 20th percentile of the combined normalized signal intensities were removed. The meta-analysis tool in NextBio uses a normalized ranking approach, which enables comparability across different gene expression datasets, platforms, and methods, independently of the absolute values of fold changes. The scoring and ranking of a gene are calculated based on the activity of the gene in each dataset and the number of datasets in which the gene is differentially expressed. Ranks are then normalized to eliminate any bias owing to varying platform size. Only genes with a *p*-value of 0.05 or less and an absolute fold-change of 1.2 or greater were regarded as significant. Pathway analysis was performed in NextBio using Gene Ontology (GO) terms and the Molecular Signatures Database (MSigDB). Gene network analysis was performed using the GeneMANIA (Warde-Farley et al., 2010), an application for gene network and pathway analyses accessed through Cytoscape v.3.0.3 (Shannon et al., 2003), another publicly available bioinformatics software. We used the default settings to include the 20 genes that have the highest number of interactions and advanced settings to include co-expression, physical, genetic, and pathways.

**Table 1:** Microarray studies in blood of PD used in meta-analysis.

GEO accession no.	No. of samples	Description	Platform	Reference
GSE54536	PD = 4; HC = 4	Untreated sporadic PD Patients (mean Hoehn and Yahr stage = 1)	Illumina HT-12 V4	Alieva et al., 2014
GSE72267	PD = 40; HC = 19	Untreated sporadic PD (mean Hoehn and Yahr stage = 1.4)	Affymetrix Human Genome U133A 2.0 Array	Calligaris et al., 2015
GSE6613	PD = 50; HC = 22	Early stage sporadic PD patients (mean Hoehn and Yahr = 2.3)	Affymetrix Human Genome U133A	Scherzer et al., 2007
GSE57475	PD = 93; HC = 49	Early stage sporadic PD, treated	Illumina HumanHT-12 V3.0	Locascio et al., 2015

## Results

To investigate whether gene expression changes in blood could provide insights into the disrupted iron metabolism in PD, we performed a meta-analysis using NextBio (Kupershmidt et al., 2010) (see Materials and Methods). Four microarrays met our inclusion criteria and were included in the final meta-analysis (**Supplementary Figure S1** and **Table S1**). Meta-analysis using the non-parametric ranking approach in NextBio identified 1,220 transcripts in blood of PD patients compared to HC. We observed very little overlap in gene expression across the four datasets. Only 24 genes were differentially expressed in at least two out of the four studies and were the most highly ranked genes (**Table 2**). The complete list of genes identified in meta-analysis is provided in **Supplementary Table S2**. The two most highly ranked genes identified in the meta-analysis were alpha synuclein (*SNCA*) and hemoglobin delta (*HBD*) (**Table 2**). Genes associated with hemoglobin, including alpha hemoglobin stabilizing protein (*AHSP*) and hemoglobin gamma 2 (*HBG2*) were downregulated in PD patients compared to HC (**Table 2** and **Supplementary Table S2**).

**Table 2:** Highly ranked genes identified in meta-analysis.

Gene symbol	Gene name	Overall gene score
*SNCA*	Synuclein, alpha (non A4 component of amyloid precursor)	198.65
*HBD*	Hemoglobin, delta	198.05
*VNN2*	Vanin 2	197.47
*LILRA5*	Leukocyte immunoglobulin-like receptor, subfamily A (with TM domain), member 5	196.91
*EPB42*	Erythrocyte membrane protein band 4.2	194.19
*MARCH8*	Membrane-associated ring finger (C3HC4) 8, E3 ubiquitin protein ligase	193.09
*DUSP6*	Dual specificity phosphatase 6	190.62
*RIOK3*	RIO kinase 3 (yeast)	185.68
*FOS*	FBJ murine osteosarcoma viral oncogene homolog	176.01
*FOSB*	FBJ murine osteosarcoma viral oncogene homolog B	165.59
*DUSP1*	Dual specificity phosphatase 1	164.85
*APBA2*	Amyloid beta (A4) precursor protein-binding, family A, member 2	163.30
*TCEA1*	Transcription elongation factor A (SII), 1	159.45
*OASL*	2′-5′-oligoadenylate synthetase-like	157.68
*XAF1*	XIAP associated factor 1	156.96
*SLC11A2*	Solute carrier family 11 (proton-coupled divalent metal ion transporters), member 2	152.44
*CA6*	Carbonic anhydrase VI	151.13
*LTBP3*	Latent transforming growth factor beta binding protein 3	146.04
*SNRNP70*	Small nuclear ribonucleoprotein 70 kDa (U1)	142.52
*LETM1*	Leucine zipper-EF-hand containing transmembrane protein 1	138.23
*NAMPT*	Nicotinamide phosphoribosyltransferase	134.16
*SSRP1*	Structure specific recognition protein 1	127.97
*TMEM19*	Transmembrane protein 19	127.97
*RPA4*	Replication protein A4, 30 kDa	127.13
*DDX17*	DEAD (Asp-Glu-Ala-Asp) box helicase 17	99.91

*Top 24 genes identified in meta-analysis. These genes were differentially expressed in at least two out of the four studies. The overall gene score is calculated from a non-parametric ranking in NextBio.*

Erythrocyte-specific genes and genes involved in heme biosynthesis and iron metabolism, including erythrocyte membrane protein band 4.2 (*EPB42*), 5′-aminolevulinate synthase 2 (*ALAS2*), solute carrier family 4 member 1 (SLC4A1), glycophorin B (*GYPB*), solute carrier family 11 member 2 (*SLC11A2*), ferrochelatase (*FECH*), and hemochromatosis (*HFE*), were downregulated in PD studies (**Supplementary Table S2**).

**FIGURE 1:**
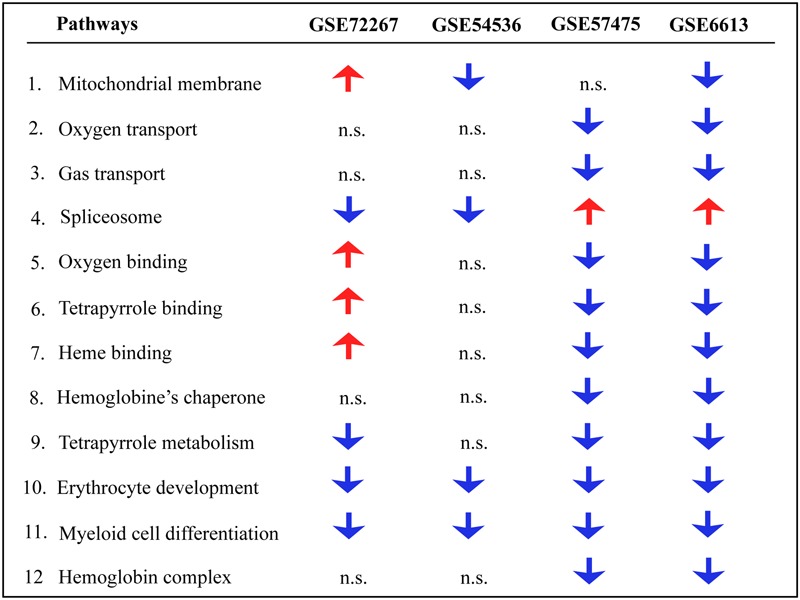
**Pathway analysis of blood microarrays in PD.** Biological and functional analysis of genes identified in the meta-analysis was performed in NextBio using the Molecular Signatures Database (MSigDB) and gene ontology terms. Red and blue arrows indicate significant overlap in upregulation and downregulation, respectively, of genes identified in the meta-analysis with the specific pathway. n.s. indicates not significant.

**FIGURE 2:**
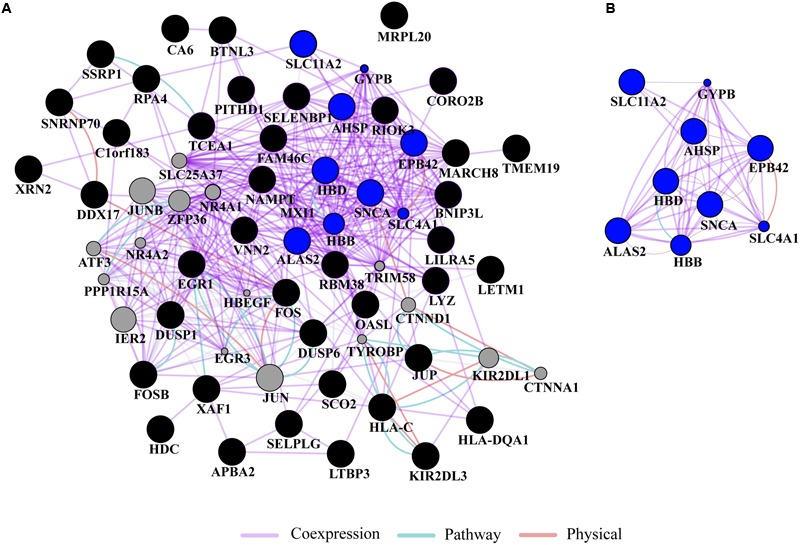
**Network analysis. (A)** Gene network analysis was performed on the top 50 most highly ranked genes identified in the meta-analysis. Input genes are shown in black circles and other genes with the greater number of interactions are displayed in gray circles. **(B)** Subnetwork of genes associated with hemoglobin and iron metabolism (blue circles) formed a highly interconnected co-expression network. The sizes of the gray nodes represent the degree of association with the input genes (i.e., smaller size represents low connectivity). Gene network analysis was performed in GeneMANIA using Cytoscape 3.0.3.

Gene ontology and pathway analysis performed in NextBio revealed that genes identified in the meta-analysis were enriched in pathways related to oxygen binding and transport, tetrapyrrole binding and metabolism, heme binding and metabolism, erythrocyte development, hemoglobin’s chaperone, hemoglobin complex, mitochondrial membrane, and the spliceosome. Genes within these pathways were predominantly downregulated across all the studies (**Figure 1**). Network analysis of the top 50 most highly ranked genes elucidated a network of genes related to hemoglobin and iron metabolism including, *HBB, HBD, AHSP, SLC11A2*, and genes involved in erythrocyte development, *EPB42* and *GYPB* (**Figure 2**). Interestingly, *SNCA* is highly interconnected within this network (**Figure 2**).

## Discussion

Microarray studies have been valuable in identifying differential gene expression patterns and perturbed biological processes in blood of PD patients (Mutez et al., 2011; Potashkin et al., 2012; Alieva et al., 2014; Calligaris et al., 2015; Santiago and Potashkin, 2015; Santiago et al., 2016; Simchovitz et al., 2016). For example, high-throughput screening of blood RNA have provided molecular clues for some of the dysregulated pathways in PD, including the impairment of insulin signaling and glucose metabolism (Santiago and Potashkin, 2013a,b, 2015), aberrant RNA splicing (Potashkin et al., 2012; Soreq et al., 2012; Alieva et al., 2014), and inflammation (Simchovitz et al., 2016). Besides these pathways, disrupted iron metabolism has been implicated in the pathogenesis of PD, but the mechanisms underlying this association remain uncertain. In this study, we utilized four independent microarray studies to investigate whether gene expression changes in blood can provide insights into the dysregulation of iron metabolism in PD patients.

Transcriptomic meta-analysis using the non-parametric ranking approach in NextBio identified several downregulated genes associated with hemoglobin and iron metabolism. Interestingly, *HBD* was the second most significant gene identified in the meta-analysis after *SNCA*. Mutations in *HBD* are associated with thalassemia, a blood disorder characterized by abnormal formation of hemoglobin resulting in disrupted oxygen transport, destruction of red blood cells, and anemia (Galanello and Origa, 2010). Hemoglobin, a protein highly expressed in red blood cells, is made up of four globulin molecules. Each globulin molecule contains an iron-containing compound called heme. Hemoglobin plays a pivotal role in oxygen transport and delivery by carrying oxygen from the lungs and delivering it to the peripheral tissues thereby maintaining cell viability (Schechter, 2008). Altered expression levels of hemoglobin in blood have been reported in several studies in PD but results are inconsistent. For instance, high levels of hemoglobin in blood of elderly men associated with an increased risk of PD (Abbott et al., 2012). Given that hemoglobin provides the most abundant source of peripheral iron, and that brain iron can be modulated by its peripheral concentration, the authors speculated that altered expression of hemoglobin in PD may be a secondary response to an ongoing iron dysregulation in the brain (Abbott et al., 2012). Conversely, low levels of hemoglobin or the presence of anemia early in life were associated with a later development of PD in both men and women (Savica et al., 2009). Strikingly, individuals who developed PD had anemia or low hemoglobin levels as early as 20 years before the onset of motor symptoms (Savica et al., 2009) suggesting that the presence of anemia or low hemoglobin levels may be one of the earliest predictors of PD. Furthermore, low levels of hemoglobin have been associated with disease severity in PD patients and late stage PD patients had lower levels of iron, ferritin, and total iron binding capacity compared to age-matched HCs (Deng et al., 2016). A recent study in a Taiwanese cohort of 86,334 patients demonstrated that newly diagnosed anemic patients have a higher risk of developing PD four or more years after the initial diagnosis of anemia (Hong et al., 2016). Thus, the mechanisms by which altered levels of hemoglobin and the presence of anemia might lead to the development of PD warrants further investigation.

Disruption of iron homeostasis has been linked to neurodegeneration in PD (Jiang et al., 2016; Matak et al., 2016). In this regard, the group of genes identified in the meta-analysis involved in iron metabolism, including *SLC11A2, ALAS2, FECH, HEBP1*, and *HFE* were downregulated in blood of PD patients. Among these genes, *SLC11A2*, previously known as *DMT1*, is the only known transmembrane transporter to be involved in cellular iron uptake and it is also required for normal hemoglobin production during erythrocyte development (Gunshin et al., 2005). A mutation in *SLC11A2* has been documented in patients with anemia and hepatic iron overload (Mims et al., 2005). In addition, dysregulation of *SLC11A2* may play a pivotal role in iron-mediated neurodegeneration in PD (Salazar et al., 2008). Another gene identified in the meta-analysis, *HFE*, was significantly downregulated in PD. Mutations in *HFE* have been associated with the development of hemochromatosis, a disease characterized with an iron overload in organs (Feder et al., 1996, 2003). Similarly, mutations in *HFE* have been documented in PD studies (Akbas et al., 2006), but the findings are inconclusive (Greco et al., 2011). *HFE* is known to modulate the levels of iron in blood (Ramos et al., 2011). Multiple blood donations, an indirect indicator of reduced iron levels, were associated with an increased risk of PD in men (Logroscino et al., 2006). Thus, the downregulation of genes associated with iron metabolism identified in meta-analysis may explain the perturbed iron homeostasis in PD.

Further, *ALAS2* and *FECH*, involved in heme and iron metabolism, have been associated with PD (Rhodes and Ritz, 2008). For example, a study confirmed the presence of a tightly correlated network of *ALAS2, FECH*, and *SNCA* in different expression datasets from human blood comprising PD samples thus proposing a molecular signature of PD (Scherzer et al., 2008). Interestingly, mutations in *SNCA* have been shown to increase cellular iron content and oxidative stress (Ostrerova-Golts et al., 2000) and iron regulatory elements are present in the 5′ UTR of *SNCA* (Friedlich et al., 2007). In our study, network analysis of the top 50 genes identified a coexpression network of downregulated genes involved in erythrocyte development, hemoglobin and iron metabolism. In the network, genes within these pathways, including *HBD, HBB, AHSP*, and *ALAS2* are connected to *SNCA*. In this context, it has been demonstrated in experiments with primates that SNCA form a complex with hemoglobin in both brain and blood and that this complex decreases mitochondrial function thus increasing the risk of PD (Yang et al., 2016). While the function of *SNCA* in blood remains unknown, several studies suggest that it may be involved in hematopoiesis, the production of cellular blood components. For example, *snca* knockout in mice resulted in hematologic abnormalities including mild anemia and smaller platelets, suggesting a potential role of *SNCA* in late stages of hematopoiesis (Xiao et al., 2014). In addition to PD, altered expression of hemoglobin genes, including *HBB, HBA1*, and *HBA2* have been found in the frontal cortex of multiple system atrophy patients, an atypical parkinsonian disorder that is often misdiagnosed as PD (Mills et al., 2016).

Genes identified in the meta-analysis were enriched in pathways related to oxygen binding and transport, tetrapyrrole binding and metabolism, heme binding and metabolism, erythrocyte development, hemoglobin’s chaperone, hemogoblin complex, mitochondrial membrane and spliceosome. In this regard, oxygen transport, heme and tetrapyrrole binding are intrinsic structural and functional properties of hemoglobin suggesting the potential loss of function of hemoglobin in PD. Further, most of these pathways were predominantly downregulated in PD patients, thus supporting the previous studies that found an association between lower levels of iron and hemoglobin in blood of PD patients.

Some of the genes identified in the meta-analysis are currently being tested as diagnostic biomarkers for PD. For example, altered expression of *SNCA* mRNA in blood has been documented in several independent studies including samples from early drug naïve PD patients (Locascio et al., 2015). Similarly, *NAMPT* was proposed as a potential diagnostic biomarker for *de novo* PD patients (Santiago et al., 2016). Notably, a combination of increased *NAMPT* mRNA in blood with hyposmia identified with the University of Pennsylvania Smell Identification Test (UPSIT-40) achieved an overall diagnostic accuracy of 86%. We expect that integration of data from molecular markers and clinical tests could significantly improve the current misdiagnosis of PD. Future studies will seek to evaluate other markers identified in this meta-analysis in blood samples obtained from drug naïve PD patients.

Collectively, the findings presented in this study provide a molecular rationale for the epidemiological studies that suggest an association between hemoglobin and iron metabolism in the pathogenesis of PD. Nevertheless, the results from this study need to be taken with caution. First, most of the hemoglobin and iron related genes were identified in datasets from PD patients receiving medication and it has been documented that drug treatment can impact gene expression in blood studies (Alieva et al., 2015). In this context, amantadine, a common drug to treat PD, may cause anemia in PD patients (Foller et al., 2008). Nonetheless, these studies alone do not explain the cases where anemia developed before PD onset (Savica et al., 2009). Future studies using gene expression datasets performed on drug naïve PD patients will be important to determine the validity of these findings. In addition, it should be emphasized that PD is a multifactorial disorder where multiple biological processes besides iron metabolism become disrupted leading to the disease pathogenesis. For instance, metabolic and neuropsychiatric disorders including, diabetes and depression, have been proposed to be risk factors for PD. Therefore, a condition of anemia alone may not put an individual at risk for PD. The altered expression of genes associated with hemoglobin, iron metabolism and anemia in blood of PD patients warrants further investigation.

## Author Contributions

Conceived and designed the experiments: JS, JP. Performed experiments: JS. Analyzed the data: JS, JP. Wrote the paper: JS, JP.

## Conflict of Interest Statement

The authors declare that the research was conducted in the absence of any commercial or financial relationships that could be construed as a potential conflict of interest.

## Footnotes

1 www.pdf.org

## Supplementary Material

FIGURE S1 **PRISMA flow chart for meta-analysis.** NextBio Research was searched for microarray studies in PD using the search terms “Parkinson’s disease (PD),” “blood,” “human,” “RNA,” and “microarray.” Four studies were identified as of August 09, 2016 that met our inclusion criteria.

TABLE S1 **PRISMA guidelines for meta-analysis**.

TABLE S2 **Meta-analysis of blood microarrays from PD patients using NextBio.** Specificity indicates the number of datasets where the gene was significantly differentially expressed. The overall gene score is calculated from a non-parametric ranking in NextBio.
