# Supplementary figures and images for: Blood Transcriptomic Meta-analysis Identifies Dysregulation of Hemoglobin and Iron Metabolism in Parkinson’ Disease
Source: Front Aging Neurosci. 2017 Mar 29;9:73. doi: 10.3389/fnagi.2017.00073 (PMC5372821; doi:10.3389/fnagi.2017.00073)

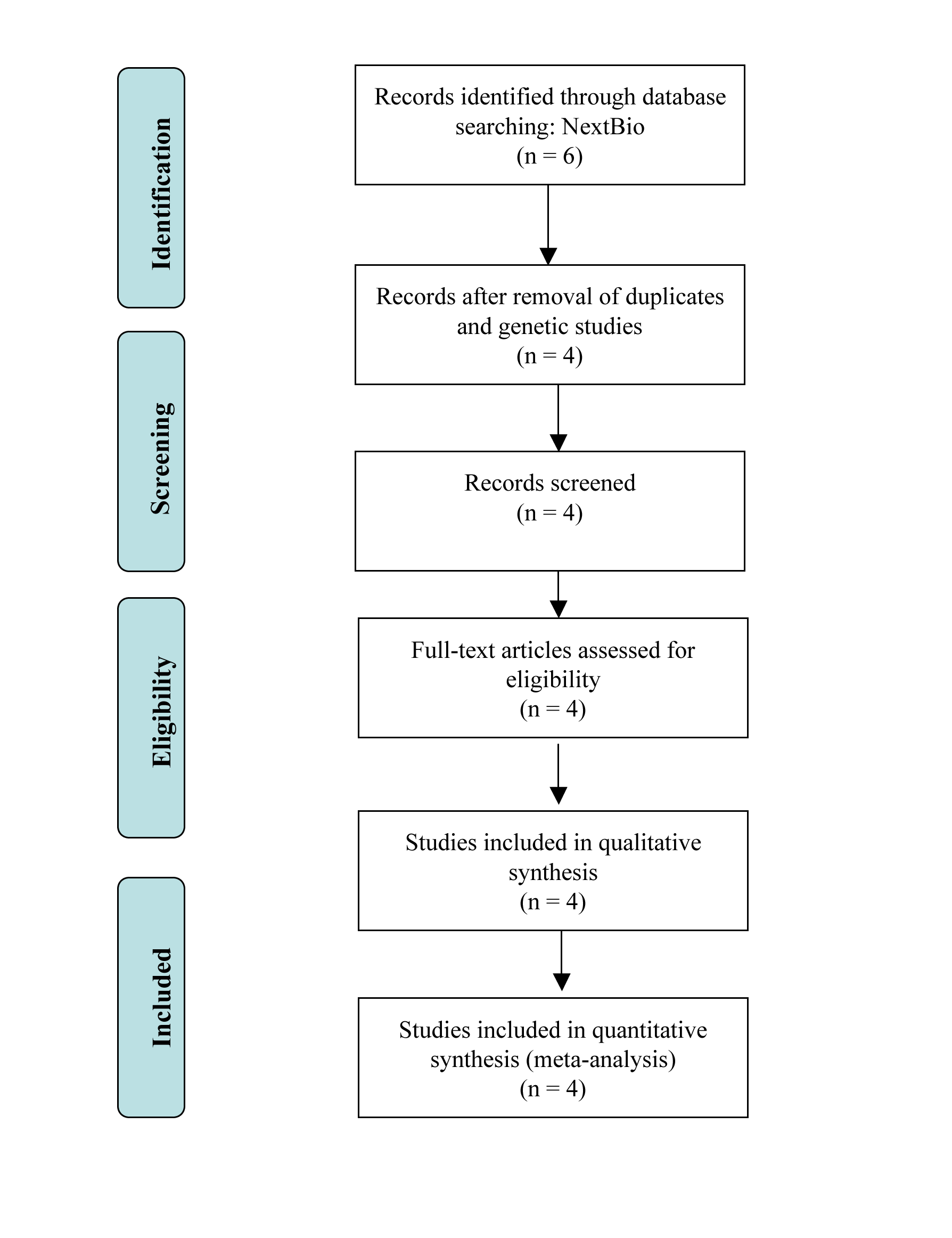

Supplement: FIGURE S1 — PRISMA flow chart for meta-analysis. NextBio Research was searched for microarray studies in PD using the search terms “Parkinson’s disease (PD),” “blood,” “human,” “RNA,” and “microarray.” Four studies were identified as of August 09, 2016 that met our inclusion criteria. [file Image_1.TIF]
